# An L-shaped flexible neural implant for chronic ECoG signal acquisition in M2 region of control and Parkinsonian rat models

**DOI:** 10.1038/s41598-025-23049-0

**Published:** 2025-11-11

**Authors:** Sreenivas Bhaskara, K. V. Shabari Girishan, Saravanan Murugaiyan, Anand Arun Dwivedi, R. Krishnakumaran, Hardik Jeetendra Pandya

**Affiliations:** 1 Department of Electronic Systems Engineering, Division of EECS, Indian Institute of Science, Bangalore, India; 2 Department of Design and Manufacturing, Division of Mechanical Sciences, Indian Institute of Science, Bangalore, India; 3 Department of Neurosurgery, Ramaiah Memorial College and Hospital, Bangalore, India; 4 Department of Bioengineering, Indian Institute of Science, Bangalore, India

**Keywords:** Flexible neural implant, Chronic signal acquisition, Parkinsonian rat model, ECoG signal, Secondary motor area, Bilateral craniotomy, Engineering, Biomedical engineering

## Abstract

Neural implants help understand neurological disorders and are actively used to study deep and cortical brain surface regions. Dealing with cortical surface regions is less complicated in clinical therapy than deep brain regions. Researchers are interested in identifying cortical surface region/s for a particular neurological disorder. Rodent models are extensively used in preclinical studies. Usually, microwires, screws, and grid-type implants are used for such studies, but they are not designed for specific rodent brain regions. Since the grids are typically standard in size, in some cases, the craniotomy required to implant the grid will be significantly bigger than the region of interest, which may pose challenges for chronic studies due to infection. Additionally, the grids may block the nearby brain regions in multisite studies, posing difficulty for another device to be implanted. In this study, a novel L-shaped surface neural implant with five electrodes (diameter: 400 μm) spanning a 1 mm × 3 mm area is fabricated using biocompatible Polyimide material for cortical surface studies. The overall thickness of the neural implant is around 25 μm. The average impedance of the electrodes is 18.315 kΩ at 1 kHz. A bilateral craniotomy is performed to place the neural implants in the secondary motor area for subdural chronic electrocorticography (ECoG) signal acquisition in control and hemi parkinsonian rats. After the recovery period, the ECoG signals are acquired using the Open BCI Cyton Daisy Biosensing board for two weeks from the rats.

**Supplementary Information:**

The online version contains supplementary material available at 10.1038/s41598-025-23049-0.

## 1. Introduction

Neural implants play a vital role in the assessment and therapeutic aspects of neurological disorders^1–4^. Cortical surface implants, known as Electrocorticography (ECoG) arrays, are commonly used in epilepsy applications to record electrophysiological signals from cortical surface regions^5^. The famous Deep Brain Stimulation electrodes have been used to treat advanced Parkinsonian patients over the decades^6,7^. The surgeries involving deep brain regions are complicated compared to those involving cortical surface regions. For example, treating advanced Parkinsonian patients involves an invasive and highly complicated neurosurgery to implant the electrodes in the deeper brain regions (e.g., subthalamic nucleus, pedunculopontine nucleus, etc.). Often, the outcomes are not fully satisfactory, and several side effects are observed^8,9^. Also, the outcomes vary depending on the target region. The stimulation of the subthalamic nucleus using invasive electrodes shows very little improvement on freezing of gait or worsens over the long run in humans^10,11^. On the other hand, repetitive transcranial magnetic stimulation of the supplementary motor area (M2) has been shown to improve freezing of gait^12–17^. The possibility of direct contact exploration of the M2 area for treating Parkinson’s disease is less explored^18,19^. Neurosurgeons often emphasize identifying cortical surface brain regions for the treatment of a disease compared to deep brain regions, as the latter involves invasive, complicated surgery. The optogenetic stimulation of the M2 area in Parkinsonian mouse models is reported^20^. However, clinical adaptation would be challenging as optical stimulation is not well adapted for treating neurological disorders. A 1 mm screw is used to record beta rhythms from an anesthetic control and lesion rat model from the M2 region^21^. However, recording electrophysiological signals from an awake subject would be more appropriate for understanding M2 role in PD. So, the electrophysiological exploration of the M2 area for Parkinson’s disease needs to be further explored. Also, whenever a new brain region needs to be explored for a particular neurological disorder, it needs to be studied in animal models, primarily rodents, before clinical trials.

**Fig. 1:**
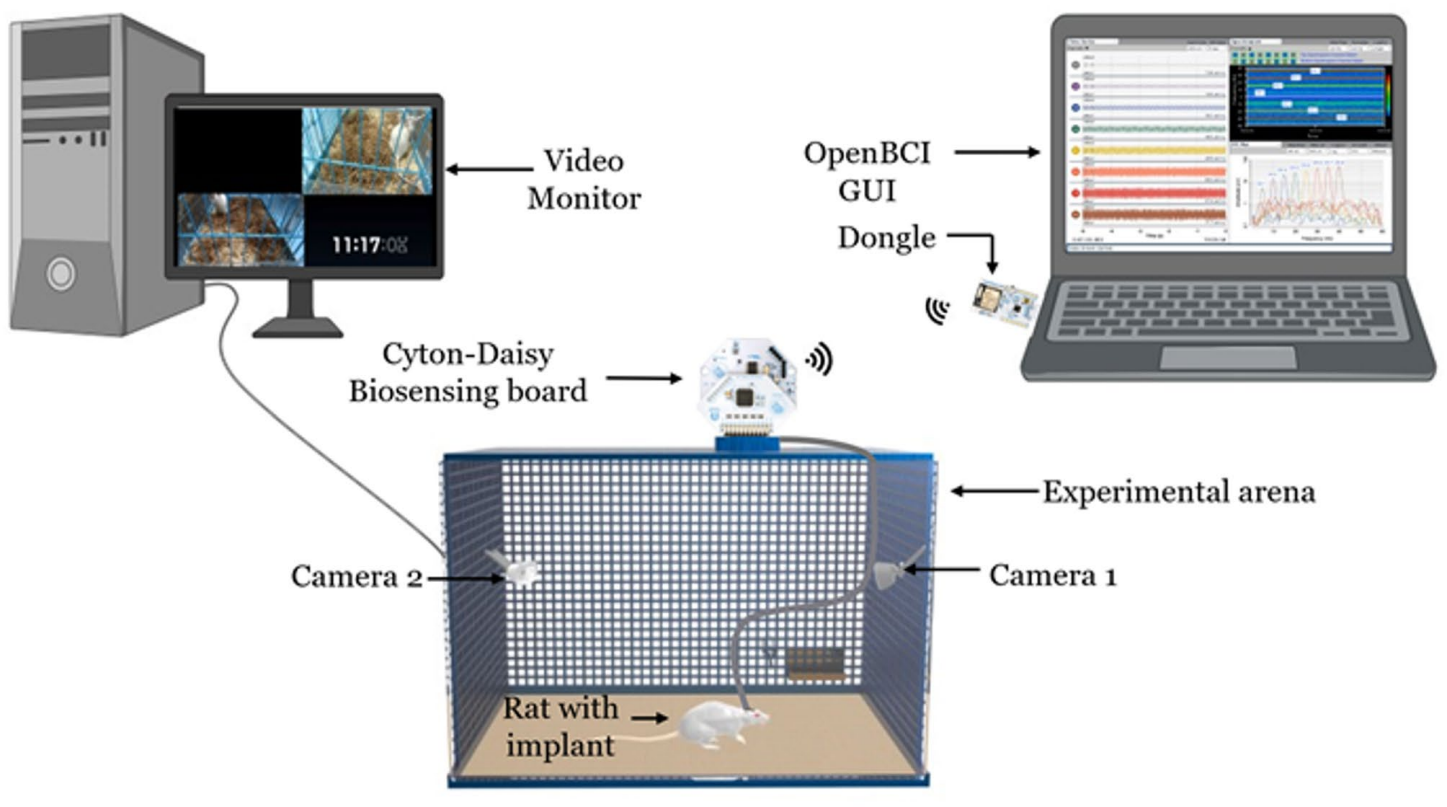
Schematic of the experimental set up for neural signal acquisition.

The researchers often use readily available, cost-effective screws and tungsten wires for signal acquisition or stimulation of motor cortex regions^22,23^. Due to the micromotion of the brain, often, screws induce damage to the brain, which in turn triggers an immune response and formation of glial scar, which would adversely affect the chronic recordings^24^. Alternately, custom-designed cortical neural implants made on biocompatible flexible substrate materials such as Polyimide, Parylene C, Polydimethylsiloxane (PDMS), etc., are used in various rodent models^25,26^. Diaz-Botia *et al*. developed a rectangular grid type of neural implant for ECoG signal acquisition^27^. Several other research groups also reported a similar structured surface neural implant with different substrates and electrode materials for applications that involve the motor cortex, somatosensory cortex, olfactory bulb, etc., in rodent models^26,28–34^. Since these surface neural implants are not optimized for a specific brain region, they are bigger than the region of interest. The bigger neural implants demand a larger craniotomy area, leading to higher chances of infections during chronic studies^35^. Alternatively, the commercially available ECoG grid arrays for rodent models are found to be expensive and are not specific to an M2 region. The above-mentioned neural implants may also impose challenges during the multisite implantation. These days, the multisite recordings in rodent models are gaining importance as the brain regions are interconnected, and decoding the connectedness is crucial for effective clinical therapies^36^. To overcome the limitations mentioned above, a flexible, implantable, L-shaped surface neural implant (SNI) with five electrodes is designed for the secondary motor cortex region (M2) of rat models and is fabricated using microfabrication techniques. The developed SNIs are characterized and implanted in control and Parkinsonian rat models for chronic neural signal acquisition, as shown in Fig. 1.

## Materials and methods

In this work, Polyimide, a flexible substrate material, is used to fabricate SNI, which is also a biocompatible material with a lower Young’s modulus than silicon^37–41^. Also, thinner and high etch resistance polyimide films can be obtained using simple spin coating and curing techniques^42^. On the other hand, PDMS suffers from metal layer adhesion, and thinner Parylene C films pose challenges during handling^26^. A noble metal, Gold (Au), is an electrode material since it is biocompatible and highly conductive^43^. The contact pad area is designed to be easily interfaced with a press-fit, low-cost, flexible printed circuit (FPC) connector. The detailed fabrication steps are provided in the later sections. After the fabrication, electrochemical impedance spectroscopy (EIS) characterization is performed before the implantation in control and Parkinsonian rat models. A unique methodology is adopted to implant SNIs on the right and left hemispheres of rat models. A base support and elevated platform are used for the implantation so that the rat cannot easily remove the electrode interface assembly after the recovery. Surface neural implants are deployed in control and Parkinsonian rat models for subdural ECoG signal acquisition for two weeks. Once the rats recover after a week, they are moved to the experimental arena for exploration and to get acquainted with it for two days. A Cyton-Daisy biosensing board is used to record the signals from both SNI (all ten electrodes). The acquisition of neural signals from the awake rat is represented in Fig. 1. A Flat Flexible Cable (FFC) is used to connect the electrode interface board to the biosensing board. Two cameras are used to record the behavior of the rat and to correlate offline with the acquired data during signal processing. Finally, the acquired data from the control and Parkinsonian rats is subjected to spectral analyses followed by comparison.

**Fig. 2:**
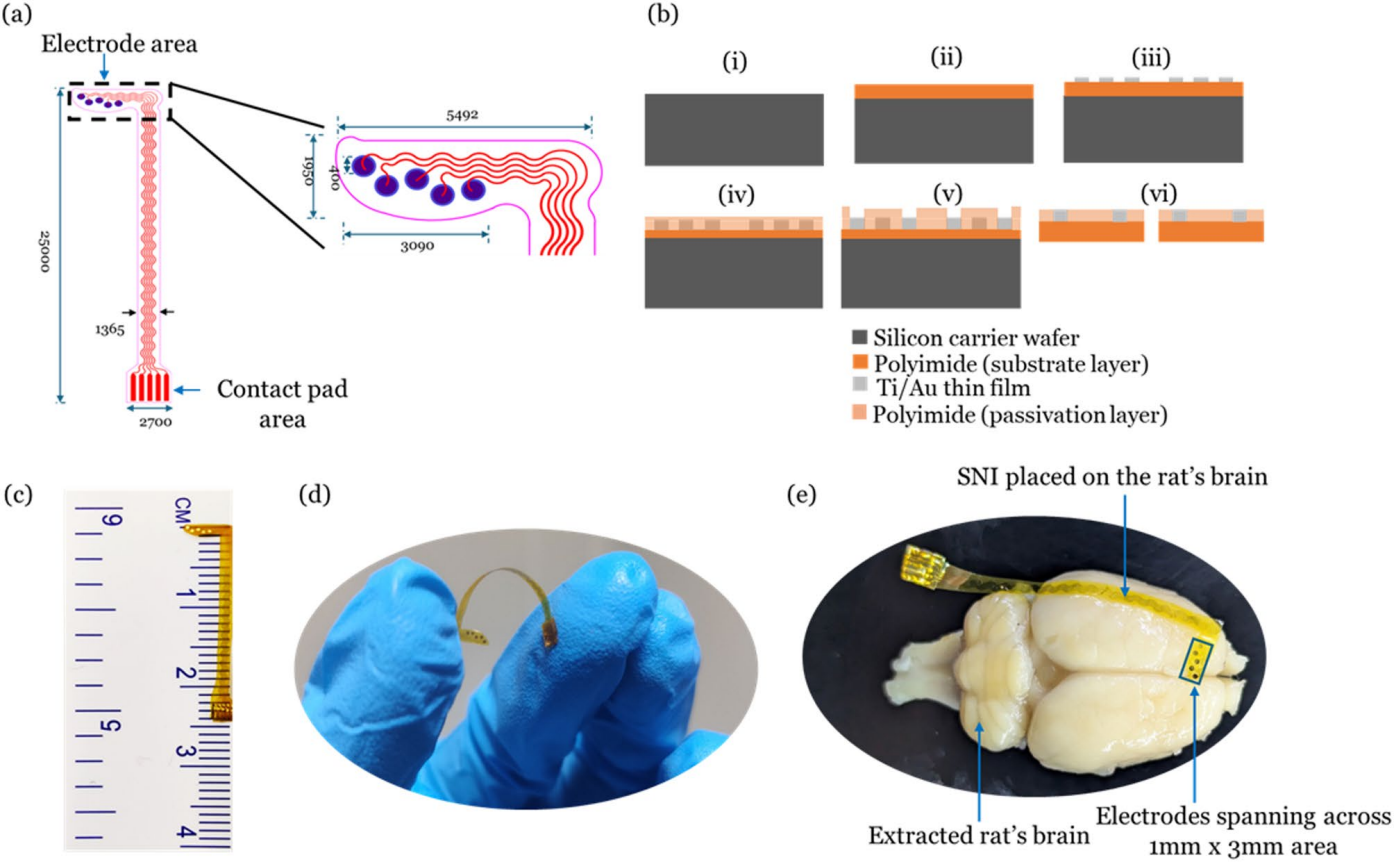
Surface neural implant design and fabrication steps: (**a**) Schematic of the implant (all measurements are in microns), (**b**) Fabrication steps: (i) Si-carrier wafer, (ii) Drop cast polyamic acid followed by spin coating and curing to form substrate Polyimide film, (iii) E-beam evaporation of Ti/Au, lithography using mask 1 followed by wet etch of Au/Ti and photoresist removal, (iv) Drop cast polyamic acid followed by spin coating and curing (passivation layer), (v) Lithography using mask 2, hard bake and subsequent dry etch of PI using plasma asher, and (vi) Devices realized from Si-wafer using a surgical blade followed by gold plating; (**c**) Fabricated SNI placed on the scale, (**d**) Photograph of the flexed SNI, and (**e**) SNI on the extracted rat’s brain.

- **Design Considerations**: The surface neural implant is designed to be placed in the secondary motor regions (M2) in the rodent brain following the anatomy of the rat brain atlas^44^. As shown in Fig. 2a, circles (blue) represent the active electrodes (diameter: 400 μm), and five electrodes are spaced at approximately 750 μm (center to center) across 3 mm laterally in a zig-zag manner. In the present work, these electrodes are used for signal acquisition. These electrodes can be used for electrical stimulation when coated with conductive polymers^45^. The diameter of the electrodes is chosen so that the electrical stimulation of cortical tissue can be possible without affecting the brain’s environment in future works^29^. The L-shape is intended to cover the M2 brain area arranged in the mediolateral orientation. The length of the implant is around 2.5 cm, so the electrode interface board (EIB) assembly can be far from the region of interest to avoid infection from external sources, which is possible in chronic studies^46^. The serpentine-shaped interconnect lines can withstand maximum strain during surgical procedures^47^. The contact pads of the SNI are designed to match the footprint of the Flexible Printed Circuit (FPC) connector on the EIB. The significant steps in the fabrication of SNI are shown in Fig. 2b.
- **Fabrication steps**: Polyimide (PI) films are obtained by curing the Poly(pyromellitic dianhydride-co-4,4′-oxydianiline) amic acid solution (Merck, United States) on a carrier wafer. All the processes required for the fabrication are carried out in a cleanroom environment (Class 100/1000). As a first step, a 3-inch dia silicon wafer (WaferPro, United States) is Piranha cleaned (Conc. H_2_SO_4_: H_2_O_2_ (3:1)(volume ratio)) for 10 min, followed by dilute Hydrofluoric acid dip (Buffer HF: DI H_2_O-18MΩ-cm (1:50)) for 30 s. The wafer is treated with low-power (48 Watts, 240 s) oxygen plasma in a plasma asher (Diener Electronic, Germany). The polyamic acid solution is drop cast onto the silicon wafer and spin-coated with acceleration (500RPM, 10 s), constant speed (1000RPM, 60 s), and deceleration (500RPM, 5 s) phases using a spin coater (MIDAS System, South Korea). The spin-coated wafer is cured in two steps (80 °C for one hour followed by 250 °C for two hours) on a hot plate (Accumax Lab Devices, India) to obtain a 20 μm thick PI layer. A Titanium adhesion layer (thickness: 10 nm) is deposited before the deposition of the Gold layer (thickness: 100 nm) using an E-beam evaporator system (Tecport, United States). The base pressure is maintained at 2*10^−6^mBar before the deposition of metals. The digital thickness monitor in the e-beam evaporation system provides the real-time thickness of the deposited metal. Before the electrode pattern creation, solvent cleaning is performed by dipping the sample in acetone (2 min), Isopropanol (2min), and DI water (1 min), respectively, followed by blow dry of N_2_ gas. A dehydration bake was performed (110 °C, 10 min) on a hot plate to remove the remaining moisture from the wafer. A positive photoresist AZ5214E (MicroChemicals GmbH, Germany) is spin-coated (500RPM (5s), 4000RPM (40s), and 500RPM (5s)) onto the wafer. Pre-exposure bake is performed on a hot plate (110 °C, 1 min). Lithography using a 4-inch photomask (Mask #1) is performed to create the metal patterns using the MJB4 Mask aligner (SUSS MicroTec SE, Germany). Mask #1 and the wafer are loaded onto the MJB4 for UV light exposure (i-line). The wafer is exposed at an intensity of 15 mW/cm^2^ for 8 s in vacuum contact mode. The wafer is unloaded and developed using AZ726MIF developer (MicroChemicals GmbH, Germany) in a petri dish for 18s. Hard baking (110 °C, 3 min) is performed before etching the Au and Ti layers using chemical etchants (Gold etchant (KI: I_2_:DI water (4 g: 1 g: 40 ml)) and Titanium etchant (mixture of ammonium hydroxide and hydrogen peroxide in the 1:2 volume ratio)). The etching time for Au and Ti is around 25 s and 9 s, respectively. The residual AZ5214E is dry etched using oxygen plasma in a Plasma asher (RF Power: 330 W, time duration: 9.5 min). Alternatively, acetone can strip off the residual AZ5214E, but caution is needed as the PI layer may peel off the silicon wafer for a longer dip in the acetone. Now, all the metal patterns on the PI layer are exposed. The electrodes and contact pads need to be exposed, and the other areas need to be passivated. The PI layer is used as a passivation layer for this implant. This step is similar to the process used to obtain a substrate polyimide layer, except that the speed in the constant phase increased to 4000RPM from 1000RPM. The curing temperature and time duration remain the same, as mentioned earlier. A passivation PI layer of 5 μm thickness is obtained in this step. A lithography step using Mask #2 is carried out to expose the electrodes and contact pad area. The wafer is solvent-cleaned before the spin coating of the photoresist. A positive resist, AZ4562, is dispensed onto the wafer and spin-coated with the spin speeds of 500, 4000, and 500 for 5 s, 40 s, and 5 s, respectively. A pre-exposure bake is performed on a hot plate to evaporate the unwanted solvents (110 °C, 1 min). The wafer and mask are loaded onto the MJB4 mask aligner before exposure using UV light (i-line). The wafer is aligned (X, Y, Theta) with the help of alignment markers concerning the alignment markers on the mask. After a thorough alignment check, the wafer is exposed at an intensity of 15 mW/cm^2^ for 12 s in vacuum contact mode. For development, the wafer is dipped into a petri dish with AZ726MIF solution and agitated slightly for 120s. Before the next step, the hard-bake of the photoresist is carried out on a hot plate (110 °C, 3 min). Finally, plasma ashing is performed (RF power: 390 W, time duration: 20 min) to etch the PI layer present over the electrode and contact pad area. No additional step is required for stripping AZ4562 as it gets etched during this ashing process. Finally, the surface neural implant (SNI) is realized by cutting the individual implant from the wafer using a surgical scalpel blade (Fig. 2c). Since the substrate is Polyimide, the flexibility can be seen in Fig. 2d. The five electrodes spanning across a 1mmx3mm area are enough to cover the secondary motor area in the rat’s brain (Fig. 2e).

**Fig. 3:**
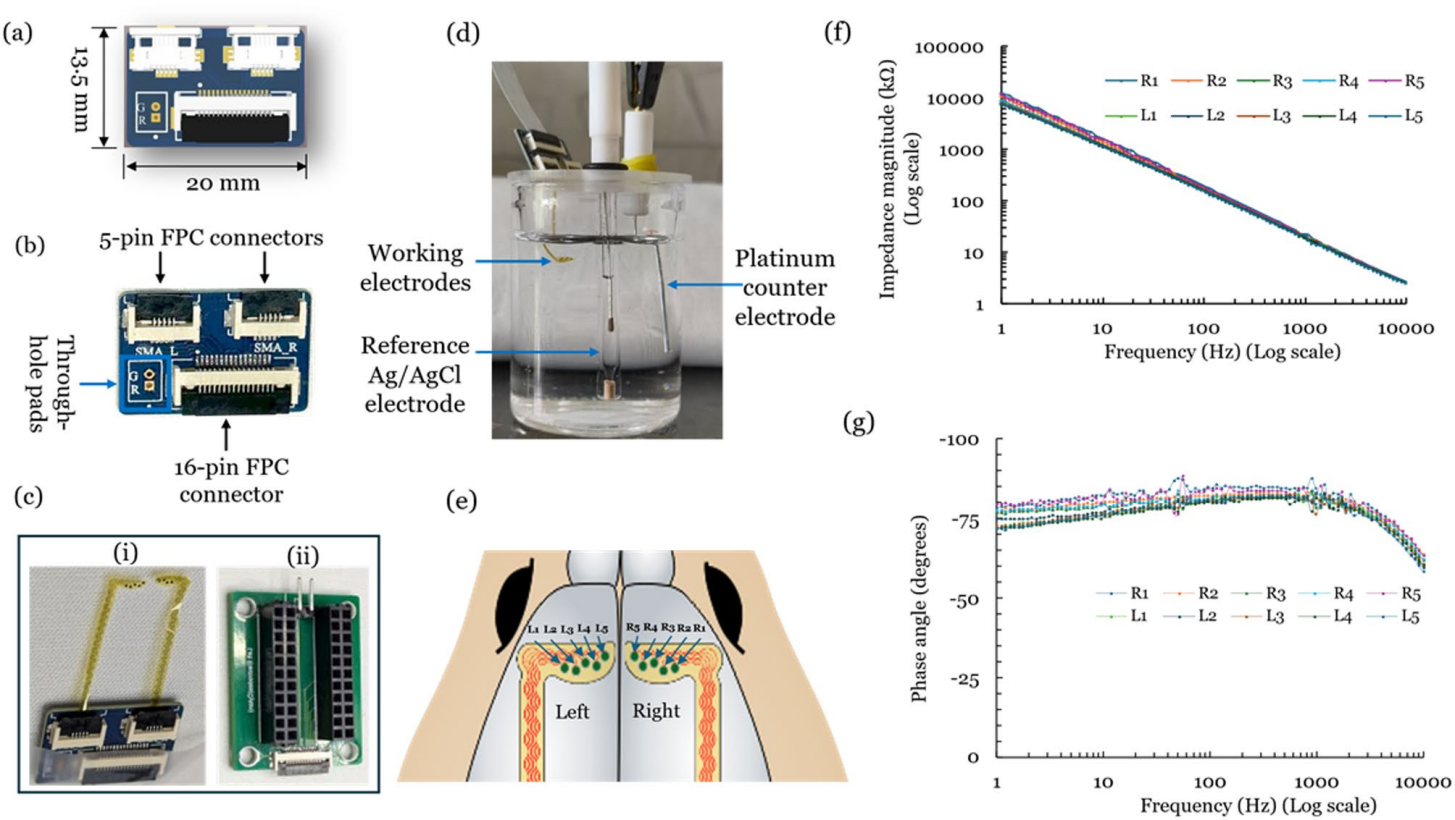
Electrode Interfacing and Characterization: (**a**) Schematic design of the custom designed electrode interface board (EIB) PCB, (**b**) EIB PCB with soldered SMT connectors, (**c**) Interfacing PCBs: (i) SNIs connected to the EIB PCB, (ii) PCB for Interfacing Cyton-Daisy Biosensing board; (**d**) Three electrode electrochemical cell set-up, (**e**) Electrode mapping schematic, (**f**) Impedance magnitude plot, and (**g**) Phase angle plot.

To further increase the thickness of the electrodes to 5 μm, gold plating is done using a Transene Sulfite Gold-TSG-25 (Cyanide-free stabilized gold sulfite) electroplating solution (Tansene Company Inc., United States). The reference and counter electrodes are shorted in the electrochemical interface. Any one of the electrodes from the SNI acts as a working electrode, and a stainless-steel metal piece of 25 mm^2^ acts as a return electrode. In accordance with the manufacturer’s recommendation, a constant current of 5.4µA for 20 min is applied between the working electrode and reference/counter electrode to obtain a thickness of 5 μm. After the fabrication, the SNIs are kept in a vacuum desiccator (Tarsons, India) to provide a dry environment. The SNIs are washed in Isopropyl alcohol (IPA-70%) before the implantation, followed by sterilization with Povidone-Iodine solution (7.5% W/V) (Win-Medicare Pvt Ltd, India). Finally, SNIs are cleaned with sterile saline before implantation.

(iii) **Surface Neural Implant Interfacing**: An Electrode Interface Board (EIB) PCB is designed using Altium Designer (Version 23). The schematic is represented in Fig. 3a. A two-layer PCB is designed with an FR-4 substrate dielectric layer and copper (top and bottom layers). The base copper thickness of the PCB is 18 μm. The track width (minimum) is kept at 0.254 mm with a minimum clearance of 0.18 mm to minimize the PCB footprint while meeting the manufacturer’s capabilities (Hi-Q Electronics, India). This EIB is designed such that on one end, two SNIs (from the left and right hemispheres of the rat’s brain) can be interfaced with the help of 5-pin FPC board connectors (0.5 mm pitch, bottom contact, front flip, Molex, United States). On the other end, a 16-pin FPC (0.5 mm pitch, bottom contact, front flip, Molex, United States) is placed to connect the acquisition system with the help of an FFC cable. A provision to interface ground and reference wire via through-hole pads can be seen in Fig. 3b. The fabricated EIB with FPC connectors soldered onto it is shown in Fig. 3b. Since the distance between the top and bottom plates of FPC connectors is more than the thickness of the SNI, the contact pad area is thickened using a bilayer stack of Kapton tape so that the press-fit mechanism works better. The SNIs are fixed to the 5-pin FPC connectors using the press-fit mechanism (Fig. 3c-i). Another PCB is designed to interface with the Cyton-Daisy biosensing board during signal acquisition, as shown in Fig. 3c-ii, and the same can also be used to access individual electrodes during characterization.
(iv) **Characterization**: Before implantation, the electrodes are characterized using electrochemical impedance spectroscopy using a frequency response analyser of Palmsens4 (PalmSens, Netherlands). Figure 3d depicts a three-electrode cell setup that uses Ag/AgCl reference and platinum counter electrodes in a 0.01 M phosphate-buffered saline (PBS, pH:7.4)^48^. Each working electrode is accessed with the help of a break-out wire connected to the interfacing PCB (Fig. 3c-ii). After connecting the respective electrodes to the terminals of the Palmsens4, an RMS voltage of 10mV is applied to the working electrode (electrode mapping is shown in Fig. 3e) with respect to the reference electrode by sweeping the frequency from 1 Hz to 10 kHz. The impedance magnitude plot of these corresponding electrodes is shown in Fig. 3f. The average impedance of these electrodes is 18.315kΩ at 1 kHz. Figure 3f, g show the impedance magnitude and phase plots, consistent with the other reported works^49,50^.

**Fig. 4:**
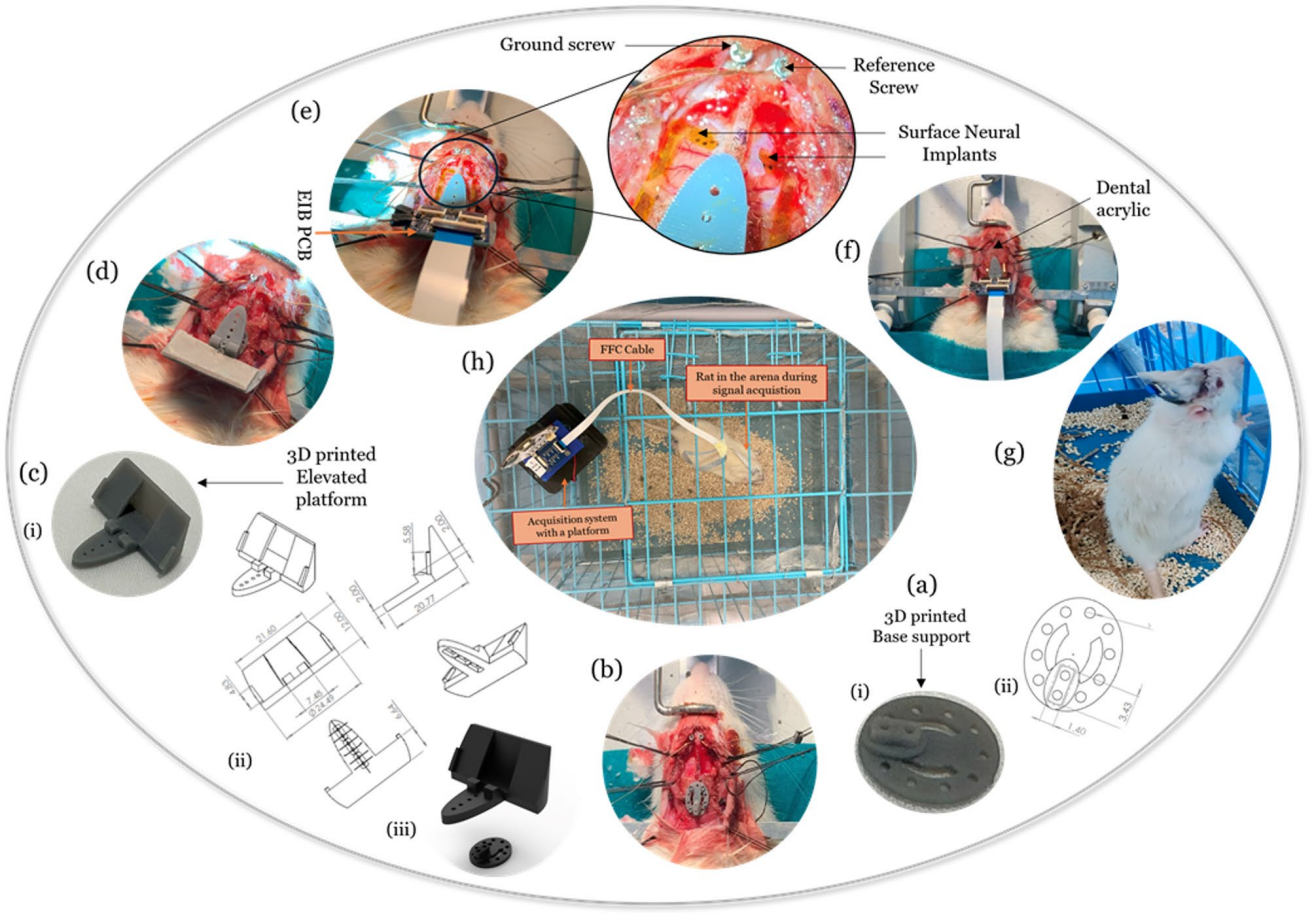
Implantation procedure and signal acquisition: (**a**) Base support: (i) Engineering drawing and (ii) 3D printed support, (**b**) Craniotomy followed by base support fixation, (**c**) Elevated platform: (i) 3D printed elevated platform, (ii) Engineering drawings of the elevated platform, (iii) Base support and elevated platform mating schematic; (**d**) Elevated platform attachment to the skull support, (**e**) EIB placement, (**f**) Layer by layer dental acrylic application, (**g**) Rat after the recovery in an experimental arena, and (**h**) Rat in an arena during signal acquisition.

**Fig. 5:**
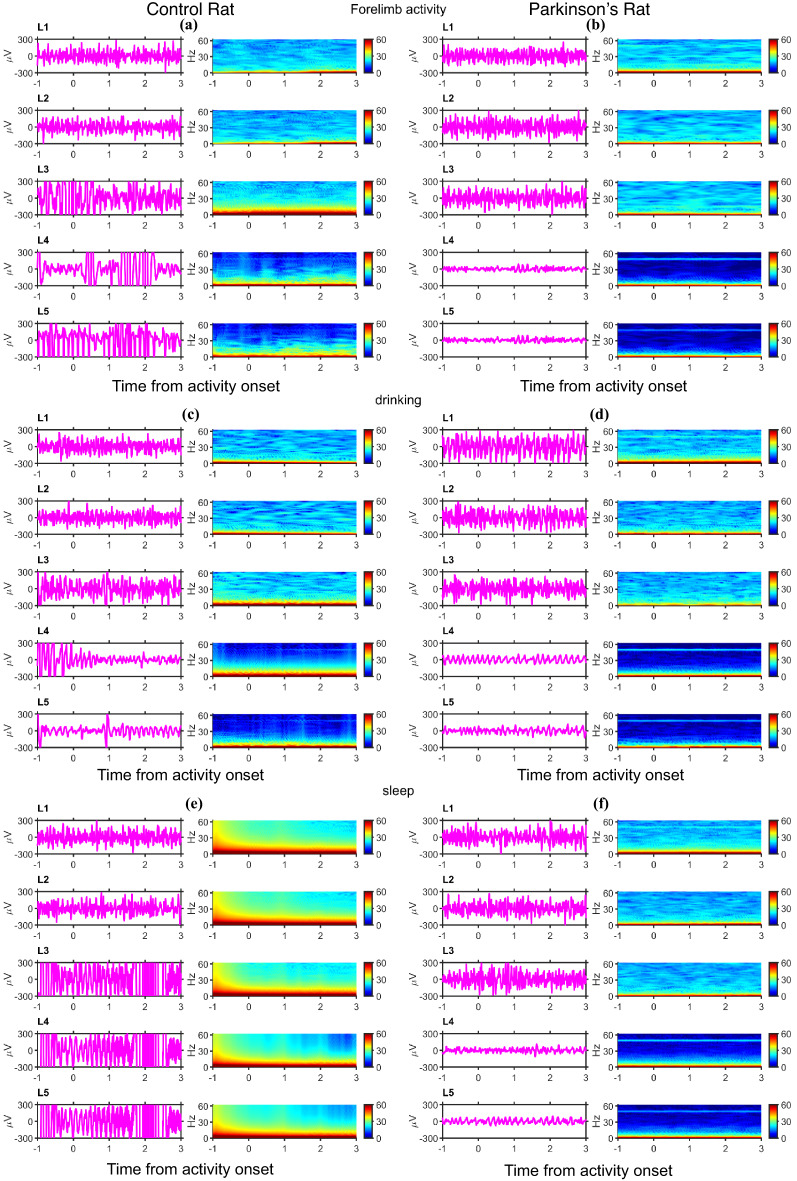
ECoG recordings from the electrodes implanted over the left hemisphere in control and PD rat (Day 12): (**a**) Example signal recorded from left hemisphere electrodes in the control rat around the onset of forelimb activity on the left column. Time Frequency (TF) spectrum averaged across all such segments on a single day in the control rat on the right column, (**b**) same as (**a**) for PD rat, (**c**) Example and mean TF spectra of signals recorded from left electrodes in control rat during drinking, (**d**) same as (**c**) for PD rat, (**e**) Example and mean TF spectra of signals recorded from left electrodes in control rat during sleep, and (**f**) same as (**e**) for PD rat.

**Fig. 6:**
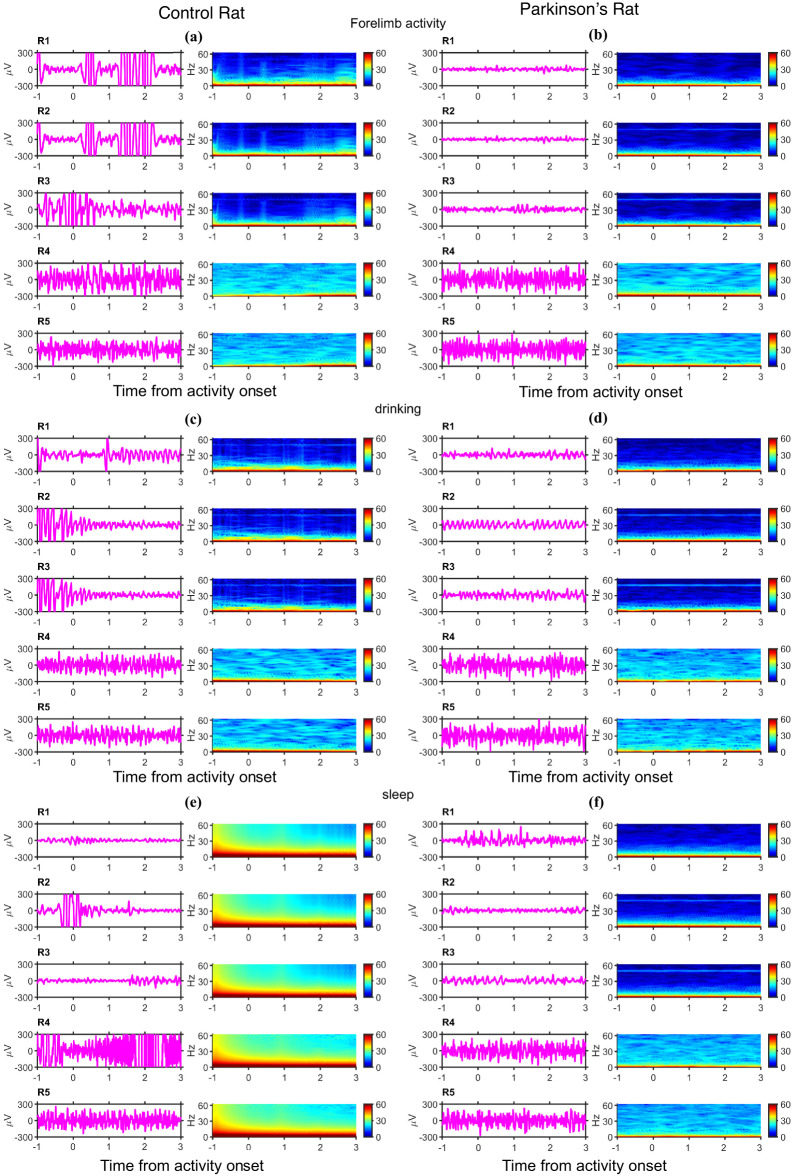
ECoG recordings from the electrodes implanted over the right hemisphere in control and PD rat (Day 12). (**a**) Example signal recorded from right hemisphere electrodes in the control rat around the onset of forelimb activity on the left column. TF spectrum averaged across all such segments on a single day in the control rat on the right column, (**b**) same as (**a**) for PD rat, (**c**) Example and mean TF spectra of signals recorded from right electrodes in control rat during drinking, (**d**) same as (**c**) for PD rat, (**e**) Example and mean TF spectra of signals recorded from right electrodes in control rat during sleep, and (**f**) same as (**e**) for PD rat.

(e) In vivo **Implantation in Control and Parkinsonian Rat Models**: All experiments were carried out in accordance with the relevant guidelines (IRB Approval No. : CAF/Ethics/090/2024, Date: October 10, 2024). The rats were purchased from Central Animal Facility at the Indian Institute of Science. The study is performed on two rats (one control and one Parkinsonian), and the implantation procedure remains the same for the control and Parkinsonian rat models. Only two rats are used for the study, as the research focuses on the performance of SNI using an in vivo rodent model.
  (i) **Implantation and neural signal acquisition (Control rat)**: A Wistar rat (*n* = 1, weight = 250 g, Male) is used as a subject for the implantation. Standard dosages of Ketamine (80 mg/kg IP) and Xylazine (10 mg/kg IP) are used for the anesthesia^51^. Behavioral reflexes are observed to check the effect of the anesthesia. The rat’s fur in the implantation region is shaved for the surgery. The anesthetized rat’s head is fixed onto a stereotaxic apparatus with the help of ear bars (RWD Life Science, China). The nose clamp and ear bars are adjusted so the rat’s head is firmly fixed to avoid any head movements. Sterile ophthalmic eye-ointment is applied with the help of sterile cotton balls on the eyes to prevent any retinal damage. The povidone-iodide solution is applied over the incision area, and the local lignocaine (7 mg/kg) is used as an analgesic. An incision is made with the help of a sterile scalpel. Excess tissues above the skull are cleared with the help of Bovie cautery (World Precision Instruments, United States) and a suction pump until the lambda and bregma points are visible. The depth of anesthesia is monitored at regular intervals, and half of the initial dosage of anesthetic drugs is provided if necessary. Two burr holes are made in the frontal region of the skull to attach ground and reference screws (1 mm diameter, 4 mm long) with microwires. The implantation area (M2 region) is marked with the help of a sterile surgical marker following the rat atlas (3.2–4.7 mm anterior, 1.5–2.0 mm lateral from bregma)^44^. A burr hole is made in the above-marked region for Intracortical microstimulation (ICMS). The ICMS is performed using a PFA-coated Tungsten wire (127 μm (conductive tip diameter), 202 μm (overall diameter), A-M systems, United States) to verify the M2 area before the implantation. The Tungsten wire is lowered to a depth of 1.8 mm with the help of the stereotaxic Z-arm and followed the stimulation protocols reported elsewhere^52^ (Stimulation parameters: cathodal first biphasic current: 30–120µA (started with 30 µA with an increment of 10µA until the clear visual response is observed), pulse frequency: 300 Hz, cathodal/anodal pulse ON time: 200µs, inter-phase delay: 100µs, pulse train duration: 40ms, frequency of the train: 1 Hz, and 10-second stimulation). A wireless brain stimulation system developed by our group is used to carry out ICMS^53^. Sometimes, it would take multiple trials to identify the correct region. It is observed that the ICMS of the primary motor cortex region (M1) connected to the forelimb elicits a visual forelimb movement, and the M2 region yields bilateral and axial muscle movements. Before the next step, the exposed brain regions are protected using sterile, haemostatic gel foam (Healthium Medtech Limited, India). A bilateral craniotomy (4 mm x 5 mm) is performed using a microdrill (0.8 mm, round tip; RWD Life Science, China). An adequate saline solution is carefully poured into the craniotomy region using a sterile syringe throughout the procedure. The debris and other fluids are cleared using a veterinary mini-suction pump (New Gen Medical System, India) with some customisation. A blunt-tip syringe needle is connected to the end of the suction pipe to get better control over the suction area. The bone flaps from the skull are secured in saline. At a later stage, these bone flaps need to be attached to the skull after the SNIs implantation. The exposed dura is protected using a sterile gel foam, as one more intermediate step is required to be done before the subdural placement of SNIs.
    An innovative implantation strategy is used to fix the electrode interface assembly. It is observed that the rats remove the electrode interface assembly once they recover from the surgery. Figure 4a-i shows the custom-designed and manufactured base support using Stereolithography (SLA) 3D printing technology (Formlabs, United States). Formlabs Grey v4.1 resin is used to print the base support and elevated platforms. The resin is not biomedical grade certified as per the manufacturer’s specifications. The bottom of the base support is coated with a thin Polyimide layer since it is in contact with the skull. After the electrodes are placed, the elevated platform is immersed (at least 50%) in dental acrylic on all sides. Figure 4a-ii depicts the engineering drawing of the base support. The thickness of the mounting rib in the base support is 1.28 mm. A small block is present above the rib (length: 3.84 mm x width:1.56 mm x height: 2.5 mm) with a provision to keep two self-tapping screws (1 mm diameter, 4 mm long). This block mates with the elevated platform in the later stage. Holes with a diameter of 0.8 mm (for screws) are provided across the periphery to fix the base support to the skull. One needs to carefully plan the place of fixation of the base support such that the electrodes of SNI will be placed in the M2 area (Fig. 4b). The elevated platform is manufactured using 3D printing technology that mates with the base support with the help of self-tapping screws (1 mm diameter, 6 mm long) (Fig. 4c-i). The engineering diagrams of the elevated platform can be seen in Fig. 4c-ii. The attachment mechanism between the base support and the elevated platform is represented in Fig. 4c-iii. The elevated platform is attached to the base support with the help of screws (Fig. 4d). Three slots are provided at the base of the elevated platform so that the position of the elevated platform can be adjusted with respect to the M2 area. This strategy offers the flexibility to adjust the SNI positions, if needed. A trial run is performed to understand which slot in the elevated platform needs to be fixed to the base support block prior to the fixation of the elevated platform to the base support. Through this trial, the position of the elevated platform will be determined. The fixation of the skull support to the mid slot of the elevated platform is usually good enough and provides a tolerance of around 3.84 mm. For subdural placement of the SNIs, the dura is cut laterally with microscissors (Fine Science Tools, United States). The EIB with SNIs is placed onto the elevated platform, and the EIB can be positioned to ensure electrodes are in the M2 region (Fig. 4e). The electrodes are pushed carefully beneath the dura with the help of precision fine-point forceps, bent at 45 degrees (Fine Science Tools, United States). The electrodes are positioned firmly with the help of a dura layer over the electrodes. The electrodes are naturally in contact with the cortex because of the fluid. The other areas of the SNIs are also attached to the skull because of the moisture on the skull. This will also ease the further steps. Once the electrodes are placed in the M2 region beneath the dura, the secured bone flaps are re-attached to the skull. The craniotomy edges are filled with small pieces of sterile gel foam to protect against leakage of dental acrylic into the cortex. Before fixation, the electrode in vivo impedances are measured using the Cyton-Daisy Biosensing Board GUI to confirm contact with the brain tissue. Our group used a low-cost Cyton-Daisy biosensing board to acquire ECoG signals earlier for rat epileptic models^54,55^. The electrode interface assembly is properly secured using dental acrylic (Fig. 4f). For subdural placement of the SNIs, the dura is cut carefully with microscissors (Fine Science Tools, United States). The EIB with SNIs is placed onto the elevated platform, and the EIB can be positioned to ensure electrodes are in the M2 region (Fig. 4e). Once the SNIs are placed in the M2 region, removed bone flaps from the M2 area are fixed back using the dental acrylic solution. Before fixation, the electrode in vivo impedances are measured using the Cyton-Daisy Biosensing Board GUI to confirm the contact with the brain tissue. The electrode interface assembly is properly secured using dental acrylic (Fig. 4f). Meloxicam is administered subcutaneously as analgesia, and Amoxicillin is administered intramuscularly as an anti-biotic for post-treatment recovery and pain management. These drugs are continued for the next 3–4 days as per the condition of the rat recovery as per the recommendations from the veterinarian. The rat is moved to its cage for recovery. During post-operative care, the animal has a liquid diet with easy access. The rat is monitored every day for the recovery. After seven days of recovery, the rat is moved to an experimental arena for neural signal acquisition (Fig. 4g). Through an FFC cable, the EIB is connected to the Cyton-Daisy biosensing board for recording neural signals from the M2 region, as shown in Fig. 4h. A similar implantation procedure is performed in a Parkinsonian rat to record the ECoG signals.
  (ii) **Hemi Parkinsonian Rat Model**: A rat is trained on a rotarod apparatus for ten days^56^ and a stepping test for four days before the Parkinson’s disease (PD) induction for motor assessment^57^. The drug 6-OHDA is popularly used for the induction of PD in naive rat models, and it has to be done through the Intracerebral route of the drug administration^58–61^. The coordinates used to induce 6-OHDA are Antero Posterior: −5.3 mm, Medio Lateral: +1.8 mm, Dorso Ventral: −7.4 mm from bregma, which is reported elsewhere^59^. A surgery is performed to create a bur hole in the above-mentioned coordinates on the right hemisphere. 6-OHDA drug is prepared 30 min before the usage as per the protocol^59^. A 10µL Hamilton syringe is used to induce the 6 µg of 6-OHDA (dissolved in 2µL of 0.2% ascorbic acid saline solution). The solution is injected with a flow rate of 0.5µL/minute for 4 min using a micro-infusion pump (World Precision Instruments, United States). The Hamilton syringe is withdrawn after 10 min of relaxation time. The bur hole closure is made using an absorbable sterile gelatin sponge. The incised skin is sutured with the help of a 4-0 Vicryl suture for recovery. Post-operative care is performed as mentioned in the earlier section. The rat is expected to develop PD symptoms on the left side of the body in 4–6 weeks. After the recovery, the rats are assessed with rotarod and stepping tests till the PD symptoms are visibly observed on the left side of the body (supplementary information: videos 1 and 2). The rat developed PD symptoms after four weeks of induction of the drug. SNI implantation and data acquisition are carried out in this PD rat, and the neural data is collected from the PD rat over two weeks. Once the data is acquired, the control and PD rats are euthanized for toxicity analysis. The specific method of euthanasia used was over-exposure to carbon dioxide in a closed chamber as per the CPCSEA-approved methods. After histopathological evaluations through an independent expert review, no visible toxicity is observed in the control or PD rats (supplementary information: Figure S1). The observations from the data acquired in control and PD rats are presented in the next section.

(f) In vivo **validation of the surface neural implants**: The Cyton-Daisy Biosensing Board acquires signals at 125 Hz with a resolution of about 0.29µV. The signal acquisition board allows measurement of in vivo impedances at around 31Hz. Also, suitable commutators for FFC cable were not available. So, an FFC cable is connected to the subject whenever there is experimentation; otherwise, the rat is set free. The experimenter would also check for any wire tangling and stop the recording immediately. The FFC cable will be removed from the rat’s head, and the wire will be uncurled before further recording. In this study, signals are recorded at a unit gain. In vivo, impedances are measured for the PD rat. For analysis, ECoG data was collected from days 9 to 13 in both control and PD cases, with the day of SNI implant surgery as ‘day 0’ for the respective subject. The time points where the rats are involved in drinking, forelimb movements, and sleep were annotated manually. Data parsing and segmenting are performed using a custom code written in Python. The remaining analyses are performed using code written in MATLAB r2022B (RRID: SCR_001622). The ECoG data recorded from 5 s before activity onset up to 10 s after each activity onset are isolated as an activity segment. To avoid movement artifacts during drinking and forelimb activity, segments overlapping periods of large wire movements are rejected from further analyses. The data collected on day 12 from each rat is shown in Figs. 5 and 6.

Figure 5 shows the data from the left hemisphere electrodes (L1 to L5), and Fig. 6 corresponds to the data from the right hemisphere electrodes (R1 to R5). In both figures, the left column presents the data from the control rat and the right column from the PD rat. Data from individual electrodes is shown in separate rows for each annotated activity. An example trace and time-frequency (TF) spectrum are shown in each row. The example traces are obtained after bandpass filtering using Butterworth (order 4) filters with a passband between 5 Hz and 40 Hz. This pre-processing provides a clear visual of the signal traces by removing slow baseline variations and high-frequency content, which constitute thermal noise. This pre-processing step is not performed for the remaining analysis. TF spectra are computed using the Multitaper method with Chronux toolbox with a time-bandwidth product of 1 over moving windows of width 0.25s sliding in steps of 0.125s^62^. The TF spectra are averaged across the corresponding activity onset segments gathered on day 12. On day 12, four segments of forelimb activity onset (Fig. 5a, b), five segments of drinking onset (Fig. 5c, d), and three segments of sleep activity are obtained with less movement-induced artifacts (Fig. 5e, f). A similar plot pattern for right hemisphere electrodes is used in Fig. 6. No significant activity onset-related signature could be isolated clearly from the TF spectra, as concurrent movement artifacts may mask these signatures. However, as is characteristic of neural signals, most power concentrations occurred at lower frequencies. At higher frequencies, some electrodes showed higher power than others. This can be attributed to thermal noise, whose power density is proportional to the impedance of the electrodes. It is also observed that certain electrodes of the PD rat (L4, L5, R2, and R3) exhibit a line noise. However, the in vivo impedances measured at 31 Hz of L4, L5, R2, and R3 are 992.8kΩ, 64.4kΩ, 234.8kΩ, and 56kΩ, respectively, during days 9 to 13, which are comparable to the impedances reported for ECoG electrodes^54^. The prominence of the line noise in these PD electrodes could be attributed to the ambient changes between the control and Parkinsonian rat sessions, which were conducted in the same experimental arena but several months apart.

**Fig. 7:**
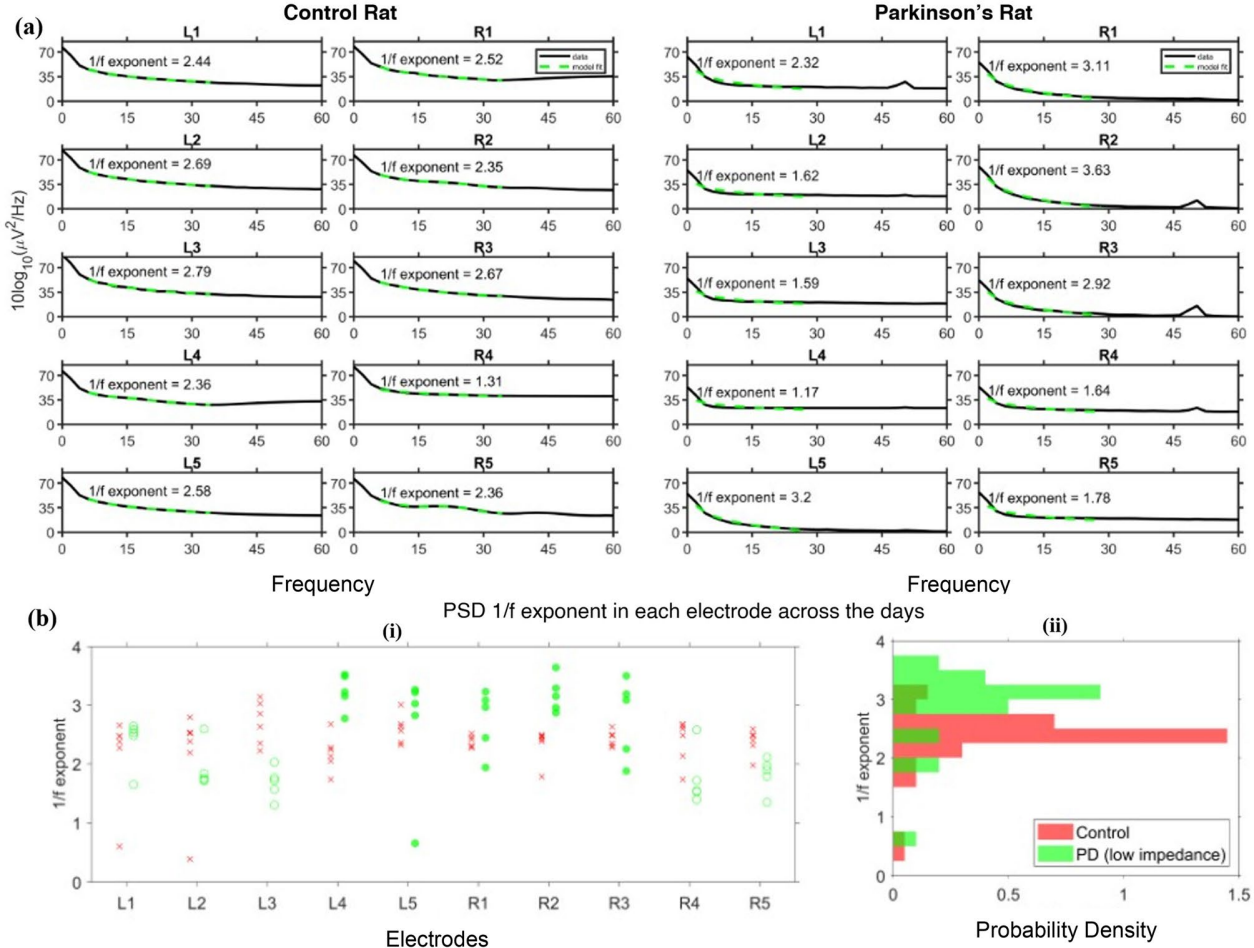
Power spectra of ECoG recordings in control and PD rats. (**a**) Mean PSD computed at each electrode on Day 12 across multiple segments of 15 s duration. FOOOF model fit of the PSD is illustrated by a green trace and the 1/f exponent estimated from the model is indicated over it, (**b**) Distribution of 1/f exponents estimated on each electrode across 5 days: (i) Exponents estimated in control rat are indicated by red ‘x’ and those in PD rat are indicated by green circles (filled circles indicate low impedance electrodes) and (ii) Histogram of 1/f exponents on different days in all electrodes in control rat (red) and low impedance electrodes in PD rat (green).

The power spectral density (PSD) is computed from data collected from five consecutive days to characterize the spectrum. On each day, non-overlapping segments of ECoG signals with a duration of 15 s, spaced at least 10 s apart, are randomly chosen throughout the day’s recording duration, ignoring recording snippets with persistent wire movements, which gives 32–48 segments per day. Each day, the average PSD across these segments is computed for each electrode using the multitaper method with a time-bandwidth product of 2. The average PSD is then fit with a FOOOF model within the frequency range of 3–40Hz^63^, which fits a 1/f-curve and Gaussian bumps to model the PSD. Gaussian bumps capture oscillatory activity, while the 1/f curve fits the aperiodic activity. Using the FOOOF fit, the exponent of the 1/f curve is estimated for each electrode on each day. The PSDs from the recordings on each electrode on day 12 are shown in Fig. 7a, along with the FOOOF fit, and the estimated 1/f exponent is indicated in Fig. 7a. Similar to the TF spectra in Figs. 5 and 6, some electrodes exhibit higher white noise, which can be seen as the asymptotic PSD value at higher frequencies. Thermal noise appears as white noise, with a constant PSD profile and higher power in electrodes with higher impedances. Aside from the thermal noise, the stereotypical 1/f profile of the aperiodic neural activity is seen. PSD of neural activity in any scale of recording (ECoG or EEG) exhibits a power-law relation ($\:\frac{1}{{f}^{\alpha\:}}$) with frequency. Under certain behavioral conditions and cognitive states, rhythms of different frequency ranges would be seen as bumps on top of the power-law spectrum, depending on the recording site. The exponent (α) of the 1/f spectrum reflects the relative activation of excitation to inhibition in the cortical tissue as the power law profile emerges from summed-up power spectra of synaptic dynamics of different receptor types.

The exponent (α) could reflect the balance of Excitation and Inhibition in the cortex. Gao et al. showed that steeper 1/f profiles (higher α) could indicate higher inhibitory activity captured by the electrode^64^. Another contribution to the 1/f power spectrum is a consequence of self-organized criticality (SOC) in the cortical system. Indeed, microelectrode recordings from cortical tissue ex vivo show evidence of neuronal avalanches^65^, as predicted by the SOC theory. In either case, it is predicted that physiological changes from aging or cognitive disorders would be reflected in the estimate of α. In humans, the exponent (α) has been found to decrease with age^66,67^, whereas, in cases of PD, α is higher, reflecting a steeper PSD profile [44,45](EEG^68,69^; Subthalamic potential^70^. The increase in steepness of the PSD profile correlates with the over-activation of inhibition brought about by dopamine depletion in PD^64^. In this study, it is indeed observed that the PD model exhibits a higher value of α than the control. Figure 7b plots the estimates of α for each electrode made across days 9–13 for both subjects. In vivo, impedance measurement in the PD rat is used to identify low-impedance electrodes (impedances under 870 kΩ at 31 Hz computed using biosensing board; represented by filled circles in Fig. 7b-i). There are five electrodes (L4, L5, R1, R2, and R3) that fall under low impedance electrode category. It is seen from the figure that these electrodes (filled circles) have a narrower distribution of α estimates than others (unfilled circles). This may be due to the lower quality of FOOOF fit on the high impedance electrodes in PD, brought about by high white noise and steep 1/f curve (larger values of α). Despite the presence of steep fall seen in PSD at lower frequencies in Fig. 7a of all electrodes suggesting high values of α, higher power white noise (constant baseline power density; equivalent to α=0) in high impedance electrodes biases the FOOOF algorithm to fit the constant baseline, yielding lower values of α on several days in these electrodes. However, in vivo impedance measurements were not made in the control rat due to contemporary hardware limitations. Hence, all electrodes were considered non-preferential for our analyses in the Control case. The estimation of slopes and analyses of time-frequency spectra and traces for event-related potentials were performed over all channels in both rats. The in vivo impedance measurements in the PD rat shed light on the potential cause of the bimodality in the slope distributions across electrodes. Hence, the robust good electrodes in the PD rat were observed specifically to note the narrower distribution of slopes, indicating reliably steeper PSD spectra in the PD rat case. In Fig. 7b-ii, distributions of α between the control rat and low-impedance electrodes identified in the PD rat are compared. The values of α recorded in low impedance electrodes in PD rats are generally higher than those estimated in the control, thus not violating observations in the literature discussed above. On the other hand, ERP analyses on the instantaneous amplitude time-series obtained using the Hilbert transform for all activities, including reach, and also on the Time Frequency amplitude spectrum. This should ensure that trial-averaging does not extinguish any beta activity due to misalignments. No prominent beta band activity changes were observable across the onset of any activity in either control or PD rat electrodes. Further, the effectiveness of using autocorrelation-based aligning of signal snippets extracted at each annotation of reach onset was highly noisy and exaggerated jitters due to noise and artefacts. Furthermore, care was taken to ensure each annotation was made with the best resolution offered by the frame rate resolution of the recording (30 fps, giving a resolution of 1/30 seconds), which is about the time period of 1 cycle of the fastest oscillatory activity within the beta band. This ensured that misalignment could result in at most one cycle of beta rhythm being affected by jitter and not highlighted in the trial average. As the results show steeper slopes of PSD spectra with less variance in electrodes with lower impedances, it can be inferred that these slopes are indeed of neural origin (Fig. 7). On the other hand, on day 13, an ultra-slow rhythmic activity is observed on a single electrode (R3) during the control rat’s sleep (supplementary information: Figure S2). The rhythm is seen to emerge within 20 min after the onset of sleep activity and is sustained throughout the sleep duration, which spans for over an hour. Using the Hilbert transform, the instantaneous frequency of the recorded signal is estimated (Figure S2(b)(ii)). The median frequency of the signal within the sleep duration is thus found to be 0.023 Hz or 1.38 cycles per minute. The origin of such rhythmic frequency variation is not known. However, this rhythm is not observed on other days in the control rat or on any day in the PD rat (More details are provided in the supplementary information). Also, the number of subjects is limited to 2 (one in each condition). In the literature, the M2 region in rat models has hardly been studied for PD using ECoG arrays targeting the M2 region. In one of the studies, a 1 mm screw is used as an electrode to record signals from the M2 area under an anesthetized condition^21^. Further animal experiments, potentially with tasks requiring task learning, must be undertaken to extensively characterise and estimate the M2 signatures and the effect of PD induction.

## Conclusion

An L-shaped flexible surface neural implant is fabricated, characterized, and implanted in control and Parkinsonian rat models to acquire and understand ECoG signals specific to the secondary motor area. A brain region-specific neural implant is fabricated to optimize the real estate required on the brain and to reduce the device burden and the consequent chances of infections. The region-specific designed neural implants demand a lower craniotomy area and facilitate multisite implantation. Polyimide, a biocompatible substrate, is used as a substrate material. After characterization using the electrochemical interface, an implantation strategy is adopted with the help of base support and an elevated platform so that the rats do not easily remove the electrode interface assembly after recovery. The data is acquired for two weeks from the rats during their sleep, forelimb movement, and while drinking. The signal analyses show the PSD slopes are different for the control and Parkinson rats. The secondary motor area can be further studied to establish a definite conclusion about its association with Parkinson’s disease. The coating of gold electrodes with a conductive polymer and stimulation studies on Parkinsonian rodent models are envisaged.

## Supplementary Information

Below is the link to the electronic supplementary material.

Supplementary Material 1

Supplementary Material 2

Supplementary Material 3

## Data Availability

The datasets used and/or analyzed during the current study are available from the corresponding author upon reasonable request.
