# Supplementary material for: An L-shaped flexible neural implant for chronic ECoG signal acquisition in M2 region of control and Parkinsonian rat models
Source: Sci Rep. 2025 Nov 11;15:39461. doi: 10.1038/s41598-025-23049-0 (PMC12606291; doi:10.1038/s41598-025-23049-0)
Supplement: Supplementary file 1 — Supplementary Material 1 [file 41598_2025_23049_MOESM1_ESM.docx]

**An L-Shaped Flexible Neural Implant for Chronic ECoG Signal Acquisition in M2 Region of Control and Parkinsonian Rat Models**

Sreenivas Bhaskara^1^, Shabari Girishan K V^3,4^, Saravanan Murugaiyan^1^, Anand Arun Dwivedi^1^, Krishnakumaran R^1^, Hardik Jeetendra Pandya^1,2*^

^1^Department of Electronic Systems Engineering, Division of EECS, Indian Institute of Science, Bangalore, India

^2^Department of Design and Manufacturing, Division of Mechanical Sciences, Indian Institute of Science, Bangalore, India

^3^Department of Neurosurgery, Ramaiah Memorial College and Hospital, Bangalore, India

^4^Department of Bioengineering, Indian Institute of Science, Bangalore, India

Supplementary Information


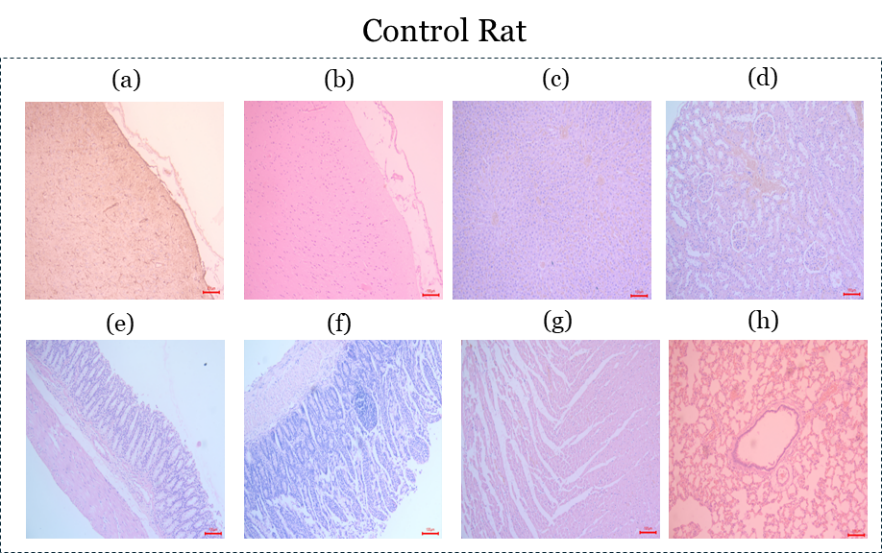

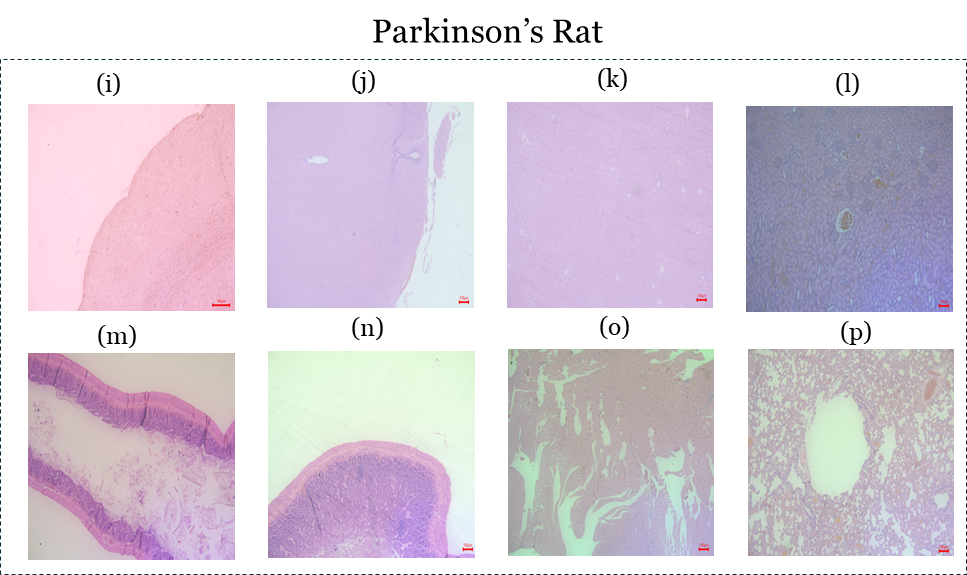


**Figure S1:** **Histopathology images (control and PD rats)** (a) GFAP staining of brain; H&E staining of slices of: (b) brain, (c) Liver, (d) Kidney, (e) Large intestine, (f) Small intestine, (g) Heart, and (h) Lungs. PD rat: (a) GFAP staining of brain; H&E staining of slices of :(b) brain, (c) Liver, (d) Kidney, (e) Large intestine, (f) Small intestine, (g) Heart, and (h) Lungs.

**Kidney:** The histology of the renal glomerulus exhibits the characteristic features of normal kidney tissues. The presence of intact kidney tubular structures with a distinct noticeable lumen, clear and undamaged glomerulus, Bowman's capsule morphologies, and visible cell nuclei indicate these results.

**Small and Large Intestine:** There were no signs of congestion, inflammation, cell death, vascular necrosis, shrinkage, excessive growth of the epithelium, excessive growth of lymphoid tissue, or abnormal growths in the small or large intestines of the rat examined.

**Heart:** The histology sections revealed a normal appearance of the heart tissue architecture, with no evidence of tissue inflammation. No changes were observed in the organization of the cardiac tissue in the sections. The sections reveal the presence of myocardium, which consists of unbroken striated heart muscle cells called cardiomyocytes. These cells have a centrally positioned oval nucleus.

**Liver:** The liver segments displayed typical hepatic characteristics on histological examination, including many hepatocytes with round euchromatic nucleoli and a sinusoidal lining devoid of cellular abnormalities. There was also no evidence of necrosis, vascular blockage, or cellular degeneration.

**Lungs:** Microscopic examination of lung sections revealed the presence of normal tissue structure in the alveolar, peribranchial, and perivascular regions, characterized by consistent alveoli and unobstructed bronchioles. The pulmonary parenchyma of the rat did not exhibit any hyperplasia or tissue necrosis.

**Brain:**

**H&E staining:** The H&E stained sections of the rat brain displayed a typical arrangement of the cerebral cortex, characterized by intact pyramidal cells with basophilic cytoplasm and conspicuous nucleoli within large rounded central nuclei. Longitudinally oriented nerve fibers were also observed between the cells, along with normal, transparent blood capillaries. There were no pyramidal cells showing necrosis.

**GFAP staining:** During the immunohistochemical analysis, the rat brain sections were stained with GFAP, which reflects the hyper-reactivity of astrocytes. The results revealed either a lack of astrocytes or a limited presence of astrocytes exhibiting positive GFAP immunoreactivity.

So, there is no visible evidence of toxicity observed in control and PD rats after few weeks of implantation of the SNI (Figure S1).

**Neural correlates of behavior in control rat**

In Figure S2, signal segments isolated about the onset of forelimb movements of the control rat across days (4 segments) with minimal movement artifacts are analyzed for behavioral correlates. In Figure S2(a), for each electrode in the control rat, mean PSDs across segments are estimated over the pre-onset (-600 to -100ms from activity onset) period and the post-onset (100 to 600ms from activity onset). In some electrodes (L5, R1, R2, and R3), a consistent increase in PSD is observed post-onset of forelimb activity after 15Hz. Typically, such broadband high-frequency increase in PSD correlates with an increase in the firing of local neurons^1,2^. Prevalent strong wire-movement-induced artifacts in the neural recordings before and during the onset of different annotated activities could not be separated from the neural correlates of the respective activities. Further, since our recording setup involved the conduction of analog neural signals via unshielded cables, the time-varying crosstalk between unshielded wires caused by bending cables prevents the cancellation of these artifacts through differential referencing.

Furthermore, on day 13, an ultra-slow rhythmic activity is observed on a single electrode (R3) during the control rat's sleep. Figure S2(b)(i) shows the signal recorded on the same day. The rhythm is seen to emerge within 20min after the onset of sleep activity and is sustained throughout the sleep duration, which spans for over an hour. Using the Hilbert transform, the instantaneous frequency of the recorded signal is estimated (Figure S2(b)(ii)). The median frequency of the signal within the sleep duration is thus found to be 0.023Hz or 1.38 cycles per minute. While minute-scale rhythms have not been reported to our knowledge, minute-scale variation of theta oscillation frequencies (just over 0.01Hz) have recently been reported during REM sleep in Local field potential (LFP) recorded from the whisker-barrel cortex of mice^3^, which the authors found is correlated with rapid eye movement onset. The origin of such rhythmic frequency variation is not known. However, this rhythm is not observed on other days in the control rat or on any day in the PD rat.

**Figure S2: Behavioral correlates in ECoG recordings from control rat.** (a) PSD of signals recorded before (black dashed line) and after (red solid line) the onset of forelimb activity averaged across all the events in all the days, (b) Sleep activity in control rat: (i) Raw signal recorded from a single electrode implanted in the control rat on day 13 (Activity of the rat is indicated by the labelled horizontal bars), (ii) Instantaneous frequency of the signal recorded during sleep (Median frequency during the second sleep period (0.0237Hz) is indicated by the magenta horizontal line).


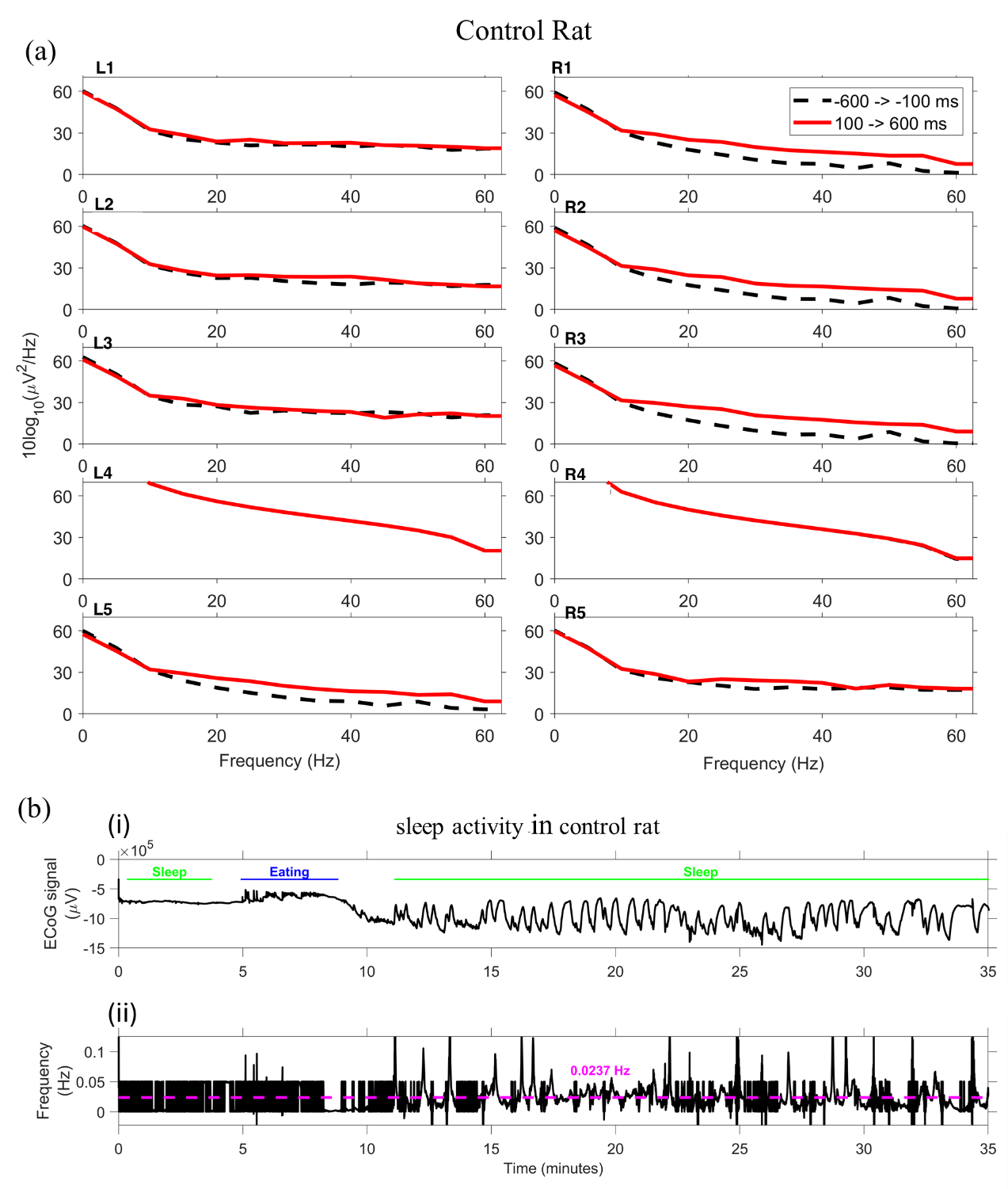


**Behavioral assessment of PD rat**

In this work, the symptoms of the hemiparkinsonian rat are assessed using the rotarod test (Video 1). During the stepping test (Video 2), it is observed that the left forelimb is not functioning properly compared to its right forelimb, indicating that the hemiparkinsonian PD symptoms are induced. Once the symptoms are observed, the SNI is implanted in the PD rat for experimentation.

**References**

1. Jia, X., Smith, M. A. & Kohn, A. Stimulus Selectivity and Spatial Coherence of Gamma Components of the Local Field Potential. *J. Neurosci.* **31**, 9390–9403 (2011).

2. Dubey, A. & Ray, S. Comparison of tuning properties of gamma and high-gamma power in local field potential (LFP) versus electrocorticogram (ECoG) in visual cortex. *Sci Rep* **10**, 5422 (2020).

3. Bueno-Junior, L. S., Ruckstuhl, M. S., Lim, M. M. & Watson, B. O. The temporal structure of REM sleep shows minute-scale fluctuations across brain and body in mice and humans. *Proceedings of the National Academy of Sciences* **120**, e2213438120 (2023).
